# A Pilot Study to Advance Task-Sharing of Gastroschisis Management in Uganda

**DOI:** 10.5334/aogh.5088

**Published:** 2026-02-25

**Authors:** Anthony N. Eze, Felix Oyania, Wigdan S. Hissein, Daphine Kyasimire, Ivan N. Nuwagaba, Gift Atuheire, OyinOluwa G Adaramola, Olivia McGinnis, Shannon Barter, Tamara N. Fitzgerald

**Affiliations:** 1 Duke University, Department of Surgery, Durham, NC, USA; 2 Duke Global Health Pathway Program, Durham, NC, USA; 3 Mbarara University of Science and Technology, Mbarara, Uganda; 4 Duke University, Durham, NC, USA; 5 Duke Global Health Institute, Durham, NC, USA

**Keywords:** global surgery, gastroschisis, midwives, nurses, task-sharing

## Abstract

*Introduction:* Gastroschisis mortality in Africa is high partly due to delays in care. In Uganda, skilled birth attendants (SBAs) are the first point-of-contact for most babies, and with proper training, may be willing to participate in surgical task-sharing.

*Objective:* Empower Ugandan skilled birth attendants with the knowledge and practical skills needed to care for babies with gastroschisis.

*Methods:* Ugandan SBAs completed a one-day gastroschisis course, and resident physicians also requested to participate. A pre- and post-course test was administered to assess gastroschisis knowledge and confidence.

*Findings:* A total of 69 SBAs (44 midwives, 25 nurses) and 17 residents participated. Participants were predominantly female (n = 64, 74%) with a median of 9 years of work experience. There was significant knowledge increase from pre- to post-course regarding differentiating gastroschisis from omphalocele (SBA 39% to 70%, p < 0.001; resident 48% to 77%, p < 0.001), gastroschisis incidence and outcomes (SBA 56% to 87%, p < 0.001; resident 65% to 89%, p < 0.001), risk factors (SBA 66% to 89%, p < 0.001; resident 67% to 86%, p < 0.0026), treatment (SBA 57% to 84%, p < 0.001; resident 63% to 79%, p < 0.001), and importance of community education (SBA 54% to 59%, p < 0.006; resident 56% to 65%, p < 0.0413). Only SBAs showed a significant increase in prenatal diagnosis (74% to 88%, p < 0.001). There was a significant boost in SBA clinical management confidence from 39% to 88%.

*Conclusion:* A one-day training course can enable Ugandan SBAs to serve as task-sharers for babies with gastroschisis. While residents benefited, a future course should be developed for their learning needs. Continuing education is needed to ensure knowledge retention and clinical preparedness. Assessment of gastroschisis outcomes is necessary to determine if involving SBAs can improve survival.

## Introduction

Less than 10% of babies with gastroschisis in sub-Saharan Africa (SSA) survive compared to a survival of 96% in high-income countries (HICs) [1–4]. While lack of total parenteral nutrition (TPN), central venous catheters, and silos is among the many factors that drive this disparity, delays in care and healthcare workforce shortages are major contributors [1]. The commercially available silo costs about $240, which is too expensive for most families, and health systems in SSA and healthcare providers often improvise [2]. Without appropriate silo application, gastroschisis mortality doubles every hour after the first 12 hours of delivery [1, 5], and previous studies have shown that many babies in SSA present to hospitals days after birth, dehydrated, with their intestines unprotected [1–4]. Recognizing the importance of silos, our team has developed a low-cost silo (<$2) using available materials in Uganda [6, 7]. This silo is pending Ugandan regulatory approval.

In Uganda, there are only 12 pediatric surgeons serving 21 million children [1, 8, 9]. There is only one pediatric surgeon in Southwestern Uganda, which is a rural and populous area. For other surgical conditions, many low-and-middle-income countries (LMICs) have successfully adopted task-sharing models that teach specific skills to health workers with less training, thereby expanding capacity and timely access to care [10–14]. Many models of task shifting and task sharing have been previously reported with a goal to improve health worker shortages, optimally utilize existing health workers, and expand access to health services [14–18]. Task sharing is the redistribution of tasks from highly specialized professionals (such as surgeons or anesthesiologists) to healthcare workers with different training (such as midwives or nurse anesthetists) [14, 19]. Task shifting moves a task entirely, whereas task sharing involves healthcare workers at different levels collaboratively participating in the same tasks [14].

In Uganda, skilled birth attendants (SBAs; midwives and nurses) represent 75% of the healthcare workforce, are widely distributed in the nation, and are the first point of contact for most Ugandan babies as 73% of babies are born under their care [20–22]. As such, Ugandan SBAs are best positioned to share in the task of delivering timely and lifesaving care to babies with gastroschisis by temporizing them prior to presenting to a pediatric surgeon. However, SBAs generally do not receive training on gastroschisis management beyond a brief introduction in school. We have previously shown that there is an overwhelming desire among Ugandan SBAs to gain supplemental training on gastroschisis management, as few feel comfortable caring for these babies without additional training [23].

In this article, we describe the design, delivery, and educational outcomes of a pilot gastroschisis management training for SBAs in Uganda, with the goal of engaging them in task sharing. While SBAs were the primary target audience, resident physicians in southwestern Uganda became aware of the course and requested to participate in this training, and were subsequently included.

## Methods

### Training course design

To design an appropriate training course that meets the needs of our target audience, we first conducted a survey and qualitative interviews elucidating the perspectives and baseline knowledge of Ugandan SBAs [23, 24]. Ugandan SBAs have a substantial interest in additional training in gastroschisis management. Nurses and midwives have similar baseline knowledge regarding gastroschisis, but there are geographical challenges and cultural implications of gastroschisis management in southwestern Uganda. SBAs prefer a training course that is offered at multiple sites, including a hands-on practical component, with educational materials, travel accommodation, and certificates of completion. Based on these findings, we designed a training course to meet the educational needs of Ugandan SBAs, while addressing common local beliefs surrounding gastroschisis.

A one-day course was designed with the following components: registration and breakfast (1 hour), pre-test assessment (45 minutes), didactics (2 hours), lunch (1 hour), hands-on silo application training using a gastroschisis simulation doll (2 hours), post-test assessment and program evaluation (1 hour), and issuing of certificates of completion (1 hour). The training was delivered in English, as preferred by the participants. A translator was available on-site to assist when needed.

**Figure 1:**
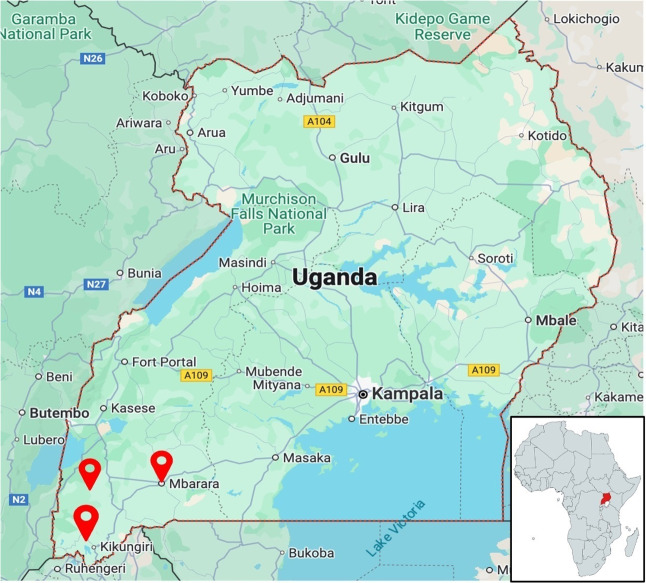
Map of Uganda; red pins indicate the training locations. The right lower corner insert shows the location of Uganda on the African continent (permission is granted from Google Maps and mapchart.net for scholarly purposes).

**Figure 2:**
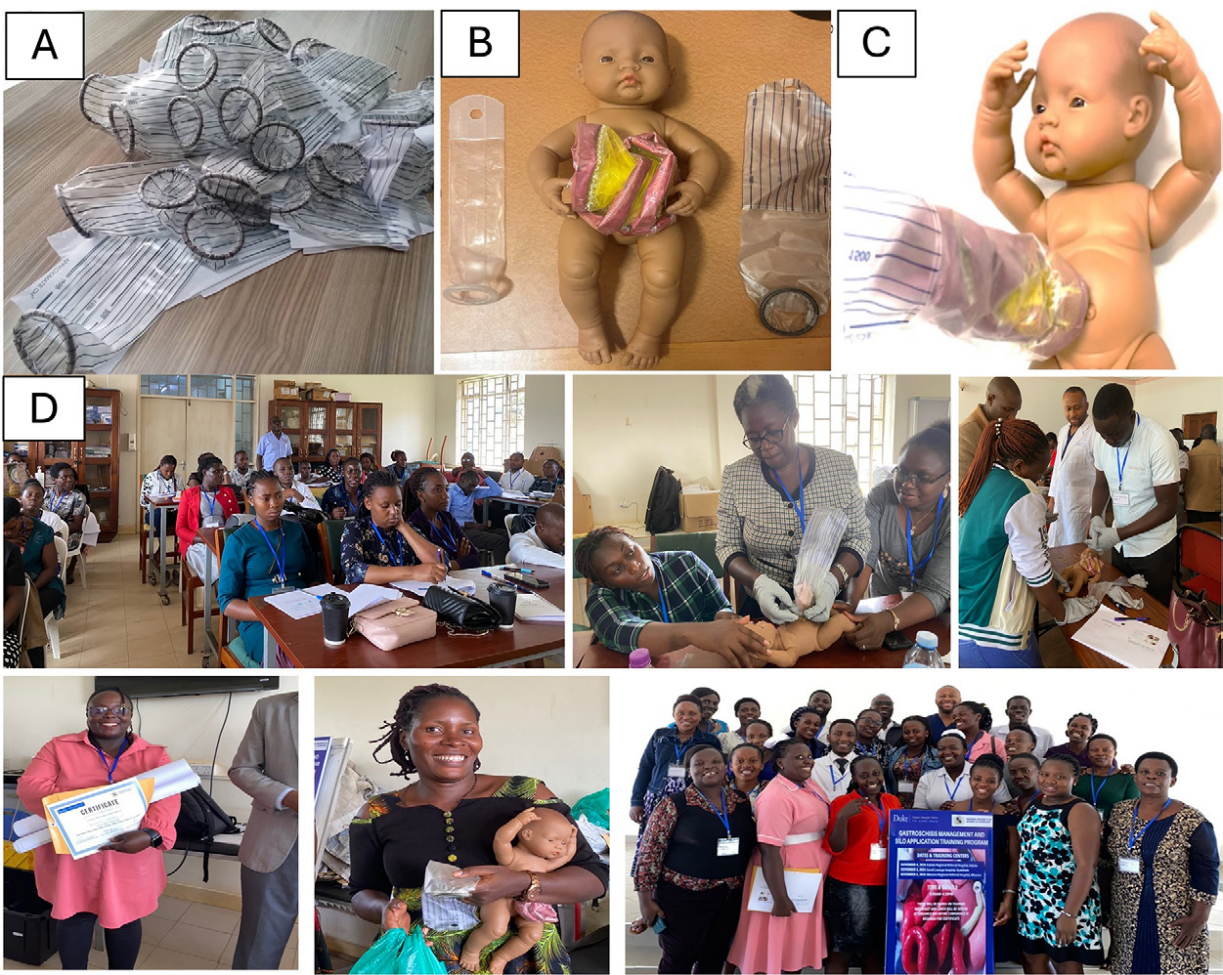
**(A)** Low-cost training silos; **(B)** low-cost gastroschisis simulation model and training silo (left: standard of care, right: low-cost silo); **(C)** low-cost gastroschisis simulation model with training silo applied; **(D)** photos from the training course depicting participants during didactics, hands-on practical with the gastroschisis simulation model and low-cost silo, and participants receiving certificates and educational materials (photos used with participant permission).

**Figure 3:**
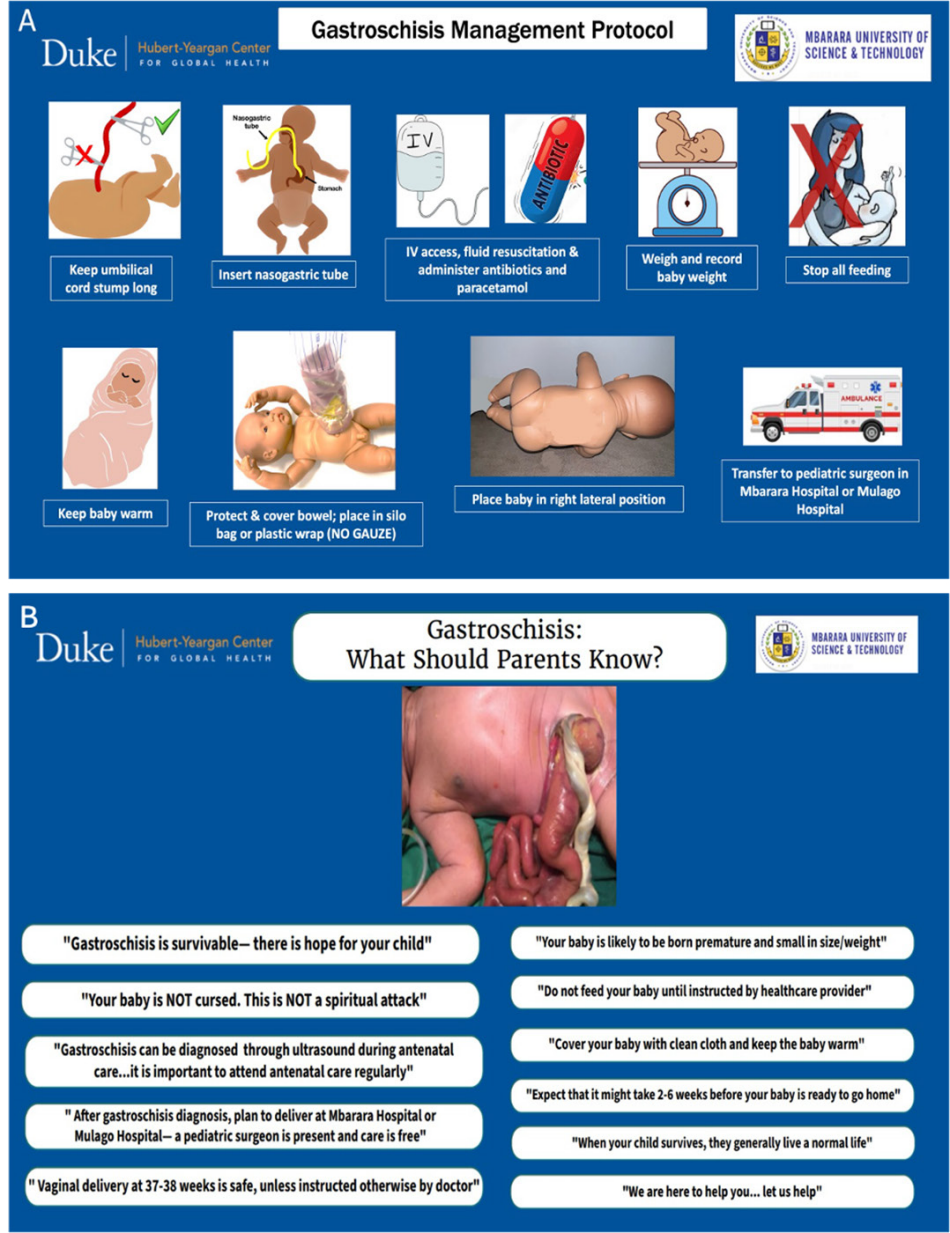
Educational posters for **(A)** healthcare providers and **(B)** parents and the community.

Three hospitals in geographic proximity to the SBAs were selected as training sites (Figure 1), with permission obtained from hospital administration. Participants were informed of their assigned training date and location four weeks in advance, allowing time to secure work coverage and administrative clearance. All participants were added to a WhatsApp community chat group for dissemination of information. The training team traveled to each location to deliver the course. All participants received compensation of 50,000 UGX ($13.64) for study participation and at least 30,000 UGX ($8.18) or more for travel reimbursement, depending on distance. For a small subset of participants who lacked access to public transportation, a vehicle was rented to ensure they could attend the course and return home on the same day, in lieu of travel compensation. Breakfast, lunch, and stationery were provided. Representatives of each health facility were gifted education materials in the form of gastroschisis simulation dolls and training silos (Figure 2A–C), educational posters (Figure 3), and educational handouts. These low-cost (<$1) training silos were purchased from our biomedical engineering team members at Makerere University, who constructed the silos using a heat-sealed urine collection bag and O-ring [6, 7]. Each participant was issued a certificate of completion by a member of the training team and a representative of the hospital leadership (Figure 2D).

### Participant recruitment and learning assessment

The Institutional Review Boards of Mbarara University of Science and Technology (MUST-2023-1207), Duke University (Pro00114298), and the Uganda National Council for Science and Technology (HS3270ES) approved the study. This study was developed in partnership with pediatric surgeons and experienced SBAs from Mbarara Regional Referral Hospital (MRRH)—the only government hospital in southwestern Uganda with pediatric surgical capacity serving patients from numerous Uganda districts and neighboring countries.

We recruited SBAs who deliver babies in southwestern Uganda, were older than 20 years of age, and were registered with the Uganda Nurses and Midwives Council. Resident physicians affiliated with course training sites were asked to participate in the course and were subsequently recruited into the study. Participation in this study was voluntary, confidential, and each participant provided a signed informed consent. All participants were issued a registration number to ensure anonymity, which they provided on all paper-based assessments and program evaluations. Participants completed a demographic survey and pre-test assessment (Appendix A). Questions assessed their knowledge regarding differentiating between gastroschisis and omphalocele, gastroschisis incidence and outcomes, prenatal diagnosis, common risk factors, treatment of gastroschisis, and the nuances of educating parents and the community about gastroschisis. These questions were developed using surgical guidelines from peer-reviewed literature, and findings from our earlier survey regarding common beliefs and perceptions in Uganda. Ugandan team members reviewed the questions for cultural appropriateness. Participants completed a post-test assessment identical to the pre-test assessment. During testing, participants were not allowed to reference materials or individuals for help. Participants also completed a program evaluation.

Data were uploaded to REDCap hosted at Duke University, to which Ugandan and US study personnel all have access [25, 26]. The negative scoring method (also known as the plus/minus method) was used for grading questions that contained more than one correct answer. The negative scoring method awards one point for each correct option but subtracts a point for each incorrect option. If the sum of points is negative, a score of zero is awarded for the question. This scoring method minimizes rewarding participants who guess [27]. A percent correct score for each category of questions and the overall test was reported.

Participant characteristics were summarized. Descriptive statistics were performed with categorical variables reported as frequencies with percentages, and continuous variables reported as medians with interquartile range (IQR). Group comparisons of participant demographics and survey responses were performed using the chi-square test and paired t-test for categorical variables as appropriate. P values < 0.05 were considered significant. Statistical analyses were performed using Stata, version 18.0 (Stata Corp, College Station, TX).

## Results

Training was delivered at three locations: Kabale Regional Referral Hospital in Kabale, Karoli Lwanga Hospital in Nyakibale, and Mbarara Regional Referral Hospital in Mbarara (Figure 1). These locations were identified as accessible to most participants. We recruited 88 participants consisting of 70 SBAs (45 midwives, 25 nurses) and 18 resident physicians (Table 1). The attrition rate was 2% as one resident and one midwife left the training early due to clinical duties. As such, both participants were excluded from analysis, yielding a final number of 86 participants who completed the training. Median participant age was 32 years [IQR 29–36]. Overall, participants were predominantly female (n = 64, 74.42%), but residents were predominantly male (n = 14, 82%, p < 0.001). SBAs were predominantly female (n = 61, 88%, p < 0.001). There were only 8 male nurses (32%) and no male midwives.

**Table 1:** Participant demographics, practice experience, and language preference.

	MIDWIFE N = 44 (51%)	NURSE N = 25 (29%)	RESIDENT N = 17 (20%)	TOTAL N = 86	P-VALUE
**Gender**					**<0.001**
Female	44 (100%)	17 (68%)	3 (18%)	64 (74%)	
Male	0 (0%)	8 (32%)	14 (82%)	22 (26%)	
**Age** (years), median [IQR]	32 [28–36]	32 [29–37]	32 [30–36]	32 [29–36]	0.391
**Level of education**					**<0.001**
Certificate- Midwifery	11 (25%)	0 (0%)	0 (0%)	11 (13%)	
Certificate- Nursing	0 (0%)	7 (28%)	0 (0%)	7 (8%)	
Diploma- Midwifery	31 (70%)	0 (0%)	0 (0%)	31 (36%)	
Diploma- Nursing	0 (0%)	15 (60%)	0 (0%)	15 (17%)	
Bachelor’s Degree- Nursing/Midwifery	2 (5%)	3 (12%)	0 (0%)	5 (6%)	
Bachelor of Medicine & Surgery (MBChB)	0 (0%)	0 (0%)	17 (100%)	17 (20%)	
**Practice experience**, median [IQR]					
Number of years worked in healthcare	10 [7–13]	9 [7–12]	5 [4–10]	9 [6–13]	0.239
Number of years delivering babies	8 [6–12]	5 [1–10]	4 [2–9]	7.5 [4–10]	0.17
Number of babies delivered per month	20 [14–30]	10 [0–20]	10 [2–30	15.5 [6–30]	0.301
Number of gastroschisis babies cared for	3 [1–4]	1 [0–4]	2 [0–4]	2 [1–4]	0.059
**Preferred language of instruction**					
English	41 (93%)	22 (88%)	16 (94%)	79 (92%)	0.735
Luganda	1 (2%)	2 (8%)	1 (6%)	4 (5%)	
Other*	2 (5%)	1 (4%)	0 (0%)	3 (3%)	

*Rukiga, Runyaankole, Kiswahili, Rutooro. Some study participants spoke multiple languages.

The most common level of education was a diploma for midwives (n = 31, 70%) and nurses (n = 15, 60%), and a Bachelor of Medicine and Surgery for resident physicians (n = 17, 100%) (Table 1). Participants had similar years of practice experience, including a median of 9 years working in healthcare, 7.5 years of which included delivering babies. Participants delivered a median of 15.5 babies per month and reported caring for a median of 2 babies presenting with gastroschisis during their career. The most preferred language of instruction was English (n = 79, 91.86%).

**Figure 4:**
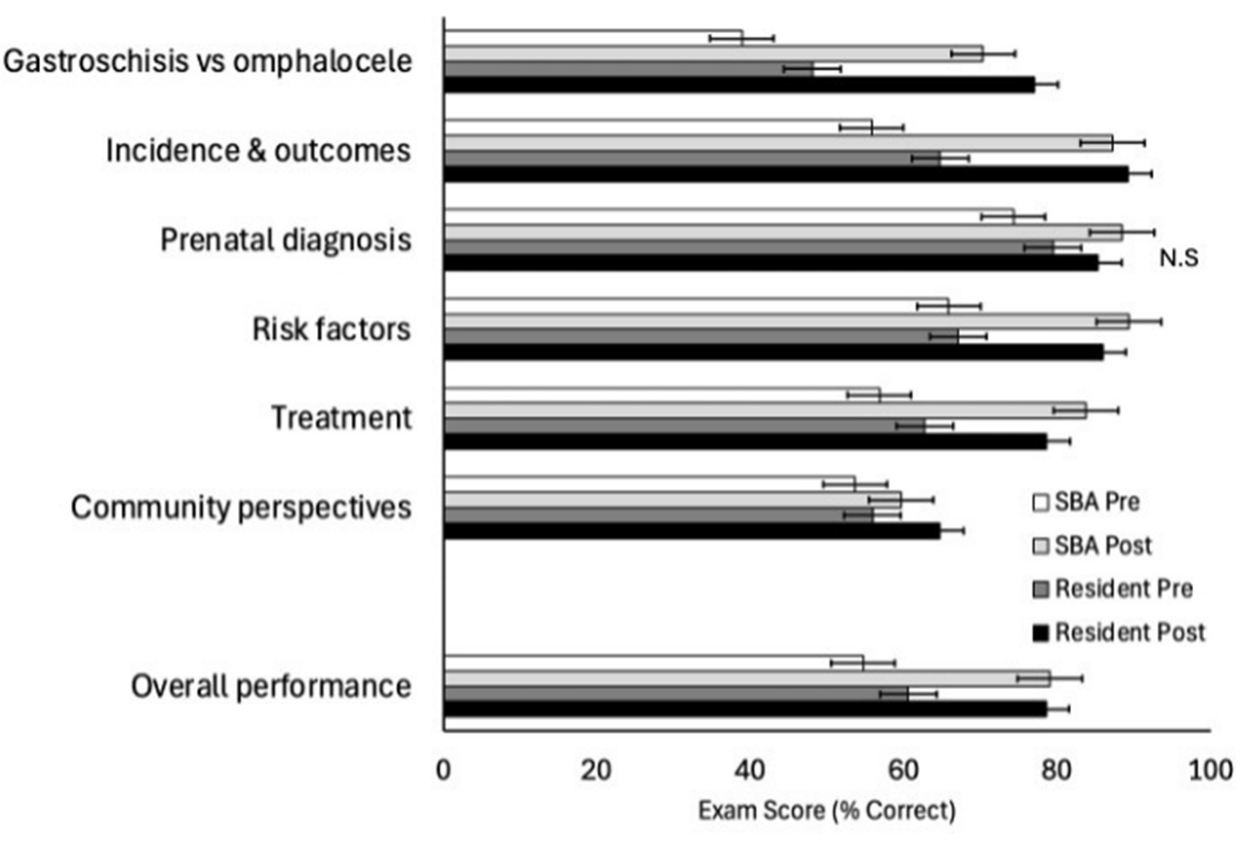
Pre- and post-course test scores for question categories and overall performance. Test scores are shown for both skilled birth attendants and resident physicians. Error bars represent standard error. All pre- and post-course comparisons demonstrated statistically significant improvement except for resident physician knowledge of prenatal diagnosis (delineated by N.S.).

After completing the training course, SBAs had a significant knowledge increase regarding differentiating between gastroschisis and omphalocele (39% pre vs 70% post; p < 0.001), gastroschisis incidence and outcomes (56% pre vs 87% post; p < 0.001), prenatal diagnosis (74% pre vs 88% post; p < 0.001), common risk factors (66% pre vs 89% post; p < 0.001), treatment of gastroschisis (57% pre vs 84% post; p < 0.001), and importance of educating the community and parents (54% pre vs 59% post; p < 0.006) (Figure 4). Similarly, there was a significant knowledge increase among resident physicians regarding differentiating between gastroschisis and omphalocele (48% pre vs 77% post; p < 0.001), gastroschisis incidence and outcomes (65% pre vs 89% post; p < 0.001), common risk factors (67% pre vs 86% post; p < 0.0026), treatment of gastroschisis (63% pre vs 79% post; p < 0.001), and importance of educating the community and parents (56% pre vs 65% post; p < 0.0413). While resident physician performance regarding prenatal diagnosis of gastroschisis trended higher after the course, this was not statistically significant (79% pre vs 85% post; p = 0.1635). Overall participant test performance was significantly higher after the course for both SBAs (55% pre vs 79% post; p < 0.001) and resident physicians (61% pre vs 79% post; p < 0.001). Among SBAs, there was a significant boost in confidence in managing babies with gastroschisis from 39% (n = 26) to 88% (n = 60) before and after the course (p < 0.001). While the proportion of resident physicians reporting confidence increased from 59% (n = 10) to 88% (n = 15), this did not reach statistical significance (p = 0.076).

**Figure 5:**
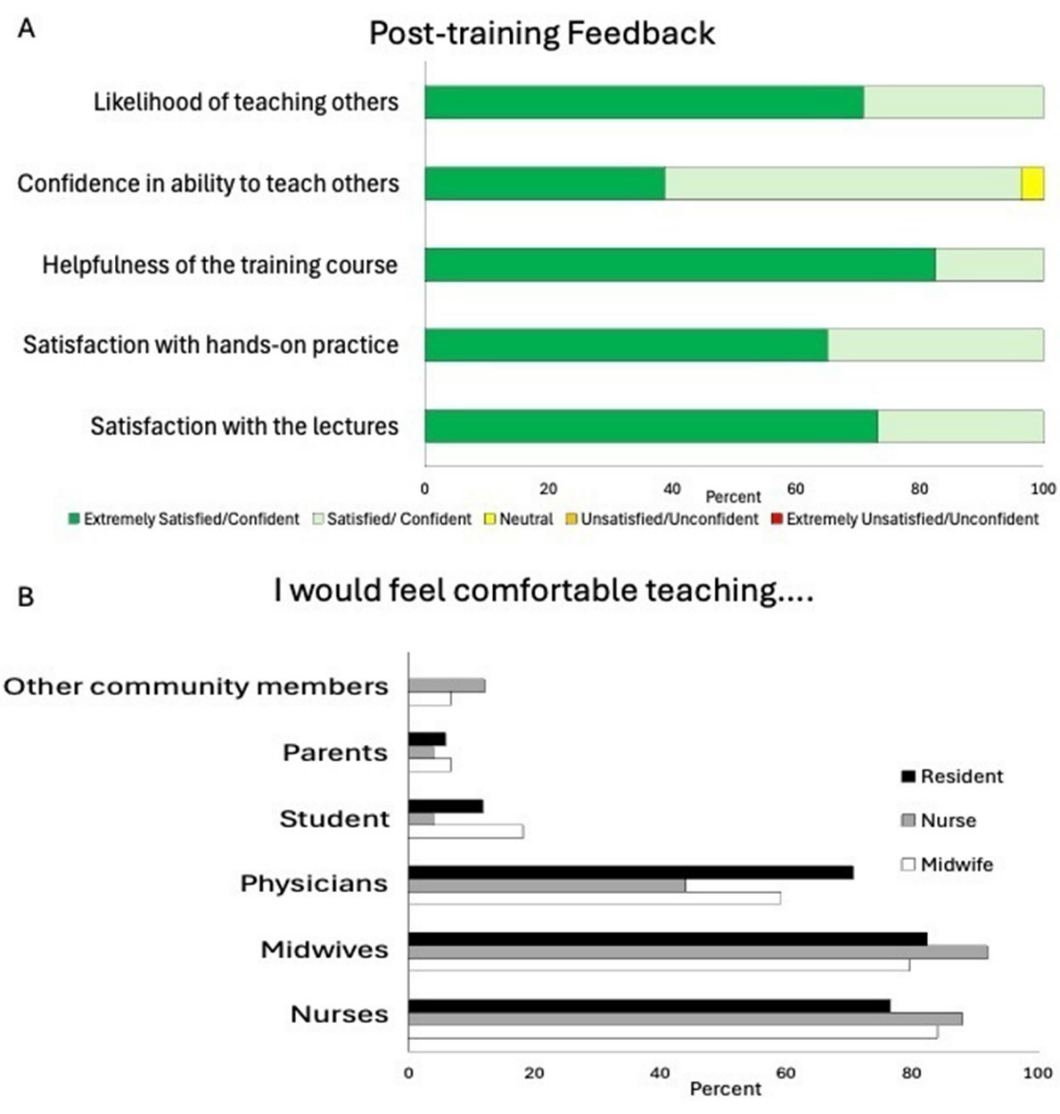
**(A)** Participant post-training feedback. **(B)** Participant comfort teaching other healthcare providers, students, and the community.

Overall, participants reported overwhelming satisfaction with the lectures/didactics (satisfied: n = 23, 27%; extremely satisfied: n = 63, 73%) and hands-on training portions of the training (satisfied: n = 30, 35%; extremely satisfied: n = 56, 65%) (Figure 5A). All participants found the training course helpful (n = 15, 17%) or extremely helpful (n = 71, 83%). All participants reported that they were likely to teach others what they had learned in the course (likely: n = 25, 29%; extremely likely n = 61, 71%), and 96% of participants reported confidence in doing so. When asked who they felt comfortable teaching, most participants preferred teaching other healthcare providers (nurses: n = 72, 84%; midwives: n = 72, 84%; physicians: n = 49, 57%) (Figure 5B). Only 13% (n = 11), 6% (n = 5), and 7% (n = 6) of participants were comfortable teaching students, parents, and other community members, respectively. There was no significant difference between the preferred audience for SBAs and resident physicians.

Participants reported that they liked the in-person mode of training delivery and provisions for geographic challenges (such as choice of training location and travel reimbursement) (n = 24, 28%), the didactic quality (n = 26, 30%), and the hands-on experience with the gastroschisis training doll and silo (n = 26, 30%). When asked what they did not like, participants reported that one day of training was too short (n = 10, 12%), and there should be more participants (n = 2, 2%). Participants suggested that the training course could be improved by offering refresher courses throughout the year (n = 10, 12%), providing follow-up support to participants to ensure they are able to teach others (n = 9, 10%), and increasing the number of participants (n = 8, 9%). Of note, only SBAs requested that the course be extended to two or more days (n = 12, 14%, p = 0.043).

## Discussion

This was a pilot study to determine the feasibility of empowering Ugandan SBAs to participate in task sharing of gastroschisis management. Task sharing is a resource management strategy adopted in many parts of Asia, the Americas, and SSA [14–17]. Task sharing has been employed in many nations in SSA wherein nurses and midwives have engaged as task sharers in health education, family planning, antenatal, and postnatal care, leading to improved maternal and child health service access and outcomes [18, 28]. Our intended audience was midwives, but while delivering the course, resident physicians expressed interest in joining and were subsequently included.

Placing exposed intestines into a silo has traditionally been a procedure that is performed by a surgeon. While components of neonatal resuscitation and referral may be within the scope of care of a midwife, performing silo application is an expansion of their practice into task sharing. This study provides initial support for a task-sharing model as demonstrated by the following: 1) participants demonstrated knowledge improvement, 2) participants reported increased confidence in clinical management, and 3) the course stimulated care coordination and improved provider communication. Going forward, potential exists for scale up to other providers and settings and future studies should be performed to expand the program and evaluate the long-term impact.

### Knowledge improvement

Previous studies from Uganda have demonstrated that midwives have increased their diagnostic accuracy in identifying congenital anomalies when they have received training and continuous professional development [29]. Our findings showed significant improvements in SBA and resident physician knowledge of gastroschisis diagnosis, treatment, risk factors, outcomes, and importance of parental and community education. As such, most participants reported high satisfaction with the training and all participants expressed a strong intention to teach other healthcare providers, students, parents, and the community. Participants were also delighted to receive donated educational resources, particularly the posters which aim to educate healthcare providers, parents, and community members on the recommended components of care for babies with gastroschisis.

We did note that resident physicians trended towards demonstrating increased knowledge in prenatal diagnosis without reaching statistical significance. This may be due to the small numbers in the study and that they entered the study with a priori education from medical school. The course was designed for SBAs, based on our previous research regarding the education needs of this population. Perhaps a future course for resident physicians should be modified to focus on their learning. Otherwise, the changes in pre- and post-training scores demonstrate that short-term knowledge acquisition among SBAs and resident physicians is achievable after a one-day training format. Similar findings were demonstrated by Skertich *et al*., wherein a simulation-based training for US general surgery residents led to improved confidence and proficiency in silo placement for gastroschisis, demonstrating the value of hands-on, scenario-based learning in skill acquisition and retention [30].

### Confidence boost and deeper learning

In addition to demonstrating increased knowledge acquisition among participants, this study showed a significant boost in SBA confidence (39% pre vs 88% post, p < 0.001) in managing babies with gastroschisis. In an earlier study from our group, only 7% of SBAs reported confidence in caring for babies with gastroschisis [23]. Resident physician confidence increased from 59% to 88%, but this did not reach statistical significance. Again, this may be due to the small number of participants, but there are also opportunities to improve the course such that all resident physicians become confident in the initial management of gastroschisis at the conclusion of a future course.

We noted during the course that meaningful discussions occurred between SBAs, resident physicians, and the instructors that deepened learning. Questions regarding initial respiratory resuscitation, nasogastric decompression, and complex clinical scenarios were common topics of conversation. Such moments revealed how training not only imparts knowledge but also actively sharpens clinical reasoning and preparedness.

### Multidisciplinary community

A WhatsApp community chat group (WhatsApp LLC, Menlo Park, CA) was initiated before the course to disseminate information on course logistics. This group included all participants and course coordinators, including the region’s only pediatric surgeon. After the course, we discovered that the chat group enabled clinical guidance, was instrumental in building a new referral system, and fostered community. Therefore, beyond the course-related benefits of the WhatsApp group, it had many clinical and health system benefits that continued after the training. Similar benefits of WhatsApp groups have previously been reported in Tanzania, where regional chat groups were used to facilitate real-time problem-solving, clinical knowledge distribution, and augment referral systems to provide tracking of cases regardless of location [31].

WhatsApp groups can provide a forum for healthcare professionals to discuss challenges, particularly in LMICs where they may be geographically isolated. The chat group from this course proved helpful as a mobile consultation platform to discuss complex patients. In one instance, an SBA consulted the pediatric surgical team regarding a baby she suspected had gastroschisis but the physician on duty was managing as a hemangioma. By providing the pediatric surgeon with a deidentified photo of the baby, he was able to diagnose the baby as a missed gastroschisis. The surgeon then reached out to the physician on duty to discuss the case and facilitate transfer.

In our earlier study, we found that patients were sometimes erroneously referred to the wrong hospital because not everyone knew that the only pediatric surgeon in the southwestern region was located at MRRH [23]. In addition to not knowing where a pediatric surgeon was located, some SBAs confused a pediatrician with a pediatric surgeon, resulting in patients being sent to the wrong regional health centers. This often led to delays in care. The WhatsApp group currently serves as a mobile referral system wherein the providers inform the pediatric surgery team about a new delivery, the current location, and expected time of arrival. Furthermore, at the time of drafting this manuscript, at least four babies with gastroschisis had silos placed shortly after birth and have been referred to MRRH using this medium. All have survived and returned home within 1–2 weeks.

Such platforms not only bridge the communication gap between distant referring facilities but also foster a sense of community and ongoing professional development. Improved communication not only helps the pediatric surgical team prepare for the baby’s arrival but also provides the referring team with feedback on the surgical outcome. Participants in our earlier study shared that due to the lack of an electronic medical record, they often did not know the outcome of patients they referred to other hospitals, leaving them wondering if they had succeeded in saving a life. For SBAs, the ability to follow up on patient outcomes via the group chat can fill a critical gap in the feedback loop, fostering reflection, professional growth, and increased confidence in further patient management and community education.

### Potential for scale up

While our training approach was initially developed for training SBAs, it has proven to be helpful for resident physician training as well. We have learned that the course could be improved by soliciting feedback from resident physicians about their educational needs and adding an additional discussion section to include complex patient scenarios. Other improvements could include extending the training duration, increasing participant numbers, and providing follow-up courses. Our training development and delivery model may also serve as a template that can be replicated and improved by teams in other nations. As others consider delivering similar trainings, we recommend developing the training by starting with an assessment of the needs and preferences of the learners.

Finally, participants were particularly delighted to use a low-cost gastroschisis simulation doll to enhance hands-on skills of bowel handling and silo application. This simulation model was specifically developed by our team for this training course in Uganda and has also been used by collaborators in Rwanda. The model can be constructed for $45 (cost of goods), is reusable, and easy to maintain [32].

### Long-term impact

Long-term knowledge retention may require regular, recurring, structured training. Many participants requested refresher courses and follow-up support to enhance their ability to deliver care and teach others. Continuing medical education would also enable providers to stay current with evolving recommendations and deepen their learning as unexpected clinical scenarios arise. Knowledge retention could also be assessed by a follow-up exam several months after the conclusion of the course.

We have given anecdotal reports of babies with gastroschisis that we believe have benefited from this course. We have observed that some babies are arriving earlier at MRRH, adequately resuscitated, with adequate bowel protection in place. However, a future clinical trial should be performed to properly assess if task sharing provides a survival benefit for gastroschisis.

## Study Limitations

The training program, while beneficial, had limitations including a small sample size and no control group, which limits the generalizability and attribution of improvements solely to the intervention. The short duration of the training and reliance on self-reported confidence also pose challenges in assessing long-term retention and actual practice changes. There are also limitations that are inherent to the current healthcare infrastructure in Uganda such as: no access to total parenteral nutrition, rare access to neonatal central venous catheters, inaccessibility and expense of silos, and workforce shortages that include inadequate numbers of pediatric surgeons, anesthesiologists, neonatologists, and neonatal nurses. Nevertheless, our team is committed to improving care for these babies as we are able [1, 2, 6, 7, 23, 24, 32].

## Recommendations

Future trainings should involve a more diverse group of healthcare workers and broader geographic regions, incorporate control groups, and conduct follow-up assessments to evaluate long-term retention and practice changes. Expansion of referral systems with structured feedback is recommended. Additionally, translating educational materials into local languages for community education will enhance the program’s scalability and sustainability.

## Conclusion

A one-day training course on gastroschisis and its management can significantly boost the Ugandan SBAs’ understanding and confidence in managing babies with gastroschisis. Continuing education will likely be needed to ensure long-term knowledge retention and clinical preparedness. Assessment of gastroschisis outcomes will be needed to determine if empowering SBAs as task sharers can improve survival.

## Figures and Tables

**Table:** 

SBA	skilled birth attendants
HIC	high-income country
LMIC	low- and middle-income country
MRRH	Mbarara Regional Referral Hospital
SSA	sub-Saharan Africa

## Additional File

The additional file for this article can be found as follows:

Supplementary Appendix A. Participant Data Collection Tool.

## References

[r1] Wesonga AS, Fitzgerald TN, Kabuye R, et al. Gastroschisis in Uganda: Opportunities for improved survival. J Pediatr Surg. 2016;51(11):1772–1777. doi:10.1016/J.JPEDSURG.2016.07.011. 27516176

[r2] Wesonga A, Situma M, Lakhoo K. Reducing gastroschisis mortality: A quality improvement initiative at a Ugandan pediatric surgery unit. World J Surg. 2020;44(5):1395–1399. doi:10.1007/s00268-020-05373-w. 31965276

[r3] Sekabira J, Hadley GP. Gastroschisis: A third world perspective. Pediatr Surg Int. 2009;25(4):327–329. doi:10.1007/S00383-009-2348-4. 19288118

[r4] Wright NJ, Sekabira J, Ade-Ajayi N. Care of infants with gastroschisis in low-resource settings. Semin Pediatr Surg. 2018;27(5):321. doi:10.1053/J.SEMPEDSURG.2018.08.004. 30413264 PMC7116007

[r5] Swift RI, Singh MP, Ziderman DA, Silverman M, Elder MA, Elder MG. A new regime in the management of gastroschisis. J Pediatr Surg. 1992;27(1):61–63. doi:10.1016/0022-3468(92)90106-H. 1532422

[r6] Leraas HJ, Biswas A, Eze A, et al. Low cost gastroschisis silo for sub-Saharan Africa: Testing in a porcine Model. World J Surg. 2023;47(2):545–551. doi:10.1007/S00268-022-06797-2. 36329222

[r7] Arivoli M, Biswas A, Burroughs N, et al. Multidisciplinary development of a low-cost gastroschisis silo for use in sub-Saharan Africa. J Surg Res. 2020;255:565–574. doi:10.1016/J.JSS.2020.05.037. 32645490

[r8] Ullrich SJ, Kakembo N, Grabski DF, et al. Burden and outcomes of neonatal surgery in Uganda: Results of a five-year prospective study. J Surg Res. 2020;246:93–99. doi:10.1016/J.JSS.2019.08.015. 31562991

[r9] Humanium. Children of Uganda. Accessed March 26, 2023. https://www.humanium.org/en/uganda/.

[r10] Rice B, Pickering A, Laurence C, et al. Emergency medicine physician supervision and mortality among patients receiving care from non-physician clinicians in a task-sharing model of emergency care in rural Uganda: A retrospective analysis of a single-centre training programme. BMJ Open. 2022;12(6):e059859. doi:10.1136/BMJOPEN-2021-059859. PMC9244677 35768107

[r11] Kinuthia R, Verani A, Gross J, et al. The development of task sharing policy and guidelines in Kenya. Hum Resour Health. 2022;20(1):61. doi:10.1186/S12960-022-00751-Y. 35906629 PMC9336004

[r12] Falk R, Taylor R, Kornelsen J, Virk R. Surgical task-sharing to non-specialist physicians in low-resource settings globally: A systematic review of the literature. World J Surg. 2020;44(5):1368–1386. doi:10.1007/S00268-019-05363-7. 31915975

[r13] Hoeft TJ, Fortney JC, Patel V, Unützer J. Task-sharing approaches to improve mental health care in rural and other low-resource settings: A systematic review. Journal of Rural Health. 2018;34(1):48–62. doi:10.1111/JRH.12229. PMC5509535 28084667

[r14] Okoroafor SC, Christmals CD. Task shifting and task sharing implementation in Africa: A scoping review on rationale and scope. Healthcare. 2023;11(8):1200. doi:10.3390/HEALTHCARE11081200. 37108033 PMC10138489

[r15] Yankam BM, Adeagbo O, Amu H, et al. Task shifting and task sharing in the health sector in sub-Saharan Africa: Evidence, success indicators, challenges, and opportunities. Pan Afr Med J. 2023;46:11. doi:10.11604/PAMJ.2023.46.11.40984. 38035152 PMC10683172

[r16] Musyimi CW, Mutiso VN, Ndetei DM, et al. Mental health treatment in Kenya: Task-sharing challenges and opportunities among informal health providers. Int J Ment Health Syst. 2017;11(1):45. doi:10.1186/S13033-017-0152-4. 28775764 PMC5540195

[r17] Dawson AJ, Buchan J, Duffield C, Homer CSE, Wijewardena K. Task shifting and sharing in maternal and reproductive health in low-income countries: A narrative synthesis of current evidence. Health Policy Plan. 2014;29(3):396–408. doi:10.1093/HEAPOL/CZT026. 23656700

[r18] Millogo T, Kouanda S, Tran NT, et al. Task sharing for family planning services, Burkina Faso. Bull World Health Organ. 2019;97(11):783–788. doi:10.2471/BLT.19.230276. 31673194 PMC6802696

[r19] World Health Organization. Task Shifting: Rational Redistribution of Tasks among Health Workforce Teams: Global Recommendations and Guidelines. WHO; 2007: 1–88. https://iris.who.int/handle/10665/43821.

[r20] Seed Global Health. Investing in nurses & midwives: Reflections from Uganda. Seed Global Health. Accessed March 26, 2023. https://seedglobalhealth.org/2020/05/12/investing-in-nurses-midwives-reflections-from-uganda/

[r21] UNMC. Provision of Quality Nursing and Midwifery Services to the Public. Accessed March 26, 2023. https://unmc.ug/.

[r22] Uganda Bureau of Statistics. Uganda Demographic and Health Survey 2016. Kampala, Uganda: UBOS and ICF; 2018.

[r23] Eze AN, Adaramola O, Kyasimire D, et al. A survey of Ugandan skilled birth attendants regarding beliefs and management of gastroschisis. BMJ Global Health-Under Review. 10.1136/bmjgh-2025-020167 PMC12983762 41802825

[r24] Adaramola OG, Eze AN, Daphine K, et al. A social-ecological model of skilled birth attendant perspectives on gastroschisis in southwest Uganda. Under Review. 2026. 10.1016/j.surg.2026.110243 42155422

[r25] Harris PA, Taylor R, Minor BL, et al. The REDCap consortium: Building an international community of software platform partners. J Biomed Inform. 2019;95:103208. doi:10.1016/J.JBI.2019.103208. 31078660 PMC7254481

[r26] Harris PA, Taylor R, Thielke R, Payne J, Gonzalez N, Conde JG. Research electronic data capture (REDCap)–a metadata-driven methodology and workflow process for providing translational research informatics support. J Biomed Inform. 2009;42(2):377–381. doi:10.1016/J.JBI.2008.08.010. 18929686 PMC2700030

[r27] Betts J, Muntean W, Kim D, Kao SC. Evaluating different scoring methods for multiple response items providing partial credit. Educ Psychol Meas. 2021;82(1):151. doi:10.1177/0013164421994636. 34992310 PMC8725057

[r28] Sakeah E, McCloskey L, Bernstein J, Yeboah-Antwi K, Mills S, Doctor HV. Can community health officer-midwives effectively integrate skilled birth attendance in the community-based health planning and services program in rural Ghana? Reprod Health. 2014;11(1):90. doi:10.1186/1742-4755-11-90. 25518900 PMC4326211

[r29] Namale-Matovu J, Kusolo R, Serunjogi R, et al. Strengthening capacity of health workers to diagnose birth defects in Ugandan hospitals from 2015 to 2021. BMC Med Educ. 2023;23(1):1–9. doi:10.1186/s12909-023-04760-w. 37833686 PMC10576368

[r30] Skertich NJ, Grunvald MW, Sullivan GA, et al. Silo placement in gastroschisis: A pilot study of simulation-based training for general surgery residents. J Pediatr Surg. 2021;56(10):1728–1731. doi:10.1016/J.JPEDSURG.2020.09.063. 33139027

[r31] Alidina S, Tibyehabwa L, Alreja SS, et al. A multimodal mentorship intervention to improve surgical quality in Tanzania’s Lake Zone: A convergent, mixed methods assessment. Hum Resour Health. 2021;19(1):115. doi:10.1186/S12960-021-00652-6. 34551758 PMC8458007

[r32] Hissein W, Eze AN, Adaramola O, et al. Development of a gastroschisis teaching model to enable surgical task sharing in Uganda. Under Review. 10.1016/j.jpedsurg.2026.163406 42628597

