# Supplementary material for: A Pilot Study to Advance Task-Sharing of Gastroschisis Management in Uganda
Source: Ann Glob Health. 2026 Feb 25;92(1):23. doi: 10.5334/aogh.5088 (PMC12947823; doi:10.5334/aogh.5088)
Supplement: Supplementary Appendix A. — Participant Data Collection Tool. [file agh-92-1-5088-s1.pdf]

Appendix A. Participant Data Collection Tool

Practice Experience

- How many years have you worked in healthcare?
- Approximately how many babies do you deliver each month?
- Approximately how many babies with gastroschisis have you delivered or cared for in your entire career?

Pre & Post Test

1. What is gastroschisis? (select one)

A congenital heart defect; An abdominal wall defect with intestines protruding; A skin condition; A neurological disorder

2. Where is the abdominal wall opening usually located in gastroschisis? (select one)

At the umbilicus; To the left of the umbilical cord; To the right of the umbilical cord; In the lower abdomen

3. Which of the following have an association with gastroschisis? (select all that apply)

Cardiac anomalies; Intestinal atresia; Chromosomal abnormalities; Neural tube defects

4. How does gastroschisis differ from omphalocele? (select all that apply)

Gastroschisis has a protective membrane covering; Omphalocele abdominal wall opening is positioned lateral to the umbilical cord; Omphalocele is associated with many other congenital anomalies; Omphalocele is associated with intestinal atresia only

5. How do you differentiate ruptured omphalocele from gastroschisis? (select all that apply)

Gastroschisis has a protective membrane covering; Gastroschisis does not have a protective membrane covering; The umbilical insertion site is key: In Omphalocele the umbilical cord and the abdominal opening are centrally located in the umbilical sac; The umbilical insertion site is key: In Omphalocele the abdominal wall opening is lateral to the umbilical cord insertion site.

6. What is the mortality rate for babies with gastroschisis in sub-Saharan Africa? (select one)

Less than 10%; Approximately 20%; About 50%; Over 90%.

7. What is the approximate incidence of gastroschisis in pregnancies? (select one)

1 in 100 to 1 in 1500; 1 in 500 to 1 in 1000; 1 in 500 to 1 in 1000; 1 in 2000 to 1 in 5000; 1 in 10,000 to 1 in 15000

8. Which of the following are true? (select all that apply)

The incidence of gastroschisis is increasing worldwide; The incidence of omphalocele is increasing worldwide; Gastroschisis co-presents with a number of other anomalies like cardiac and chromosomal abnormalities; Gastroschisis occasionally co-presents with intestinal atresia.

9. Who is typically the first point of contact for births in Uganda? (select one)

Pediatric surgeons; General practitioners; Nurses and midwives; Obstetricians

10. What prenatal/antenatal screening should diagnose gastroschisis? (select one)

CT scan; Genetic testing; Ultrasound; Amniocentesis

11. What key feature is identified on ultrasound for gastroschisis? (select one)

Presence of a membranous sac covering; Extra fingers and toes; Absence of a membranous sac covering; Normal organ placement

12. Which of the following is NOT a cultural belief surrounding gastroschisis in some communities? (select all that apply)

It is a blessing; It is a result of maternal sin/curse; It is caused by witchcraft; It is a sign of genetic superiority; It is caused by infidelity/adultery

13. What maternal factor is a significant risk for gastroschisis? (select one)

High BMI; Advanced maternal age; Young maternal age; Frequent prenatal visits; Radiation from too many ultrasounds or CT scans

14. What is a protective factor against gastroschisis? (select one)

Low fruit intake; Adequate folic acid supplementation; Smoking; Advanced maternal age

15. What should be done immediately after a baby with gastroschisis is born? (select one)

Start oral feeding; Cover the exposed bowel; Begin surgery; Administer vaccinations

16. What is the purpose of a nasogastric tube in immediate management for a baby with gastroschisis? (select all that apply)

To feed the baby; To decompress the bowel; To monitor oxygen levels; To provide hydration; To avoid aspiration

17. What is the recommended mode of delivery for a baby with gastroschisis? (select one)

Cesarean section always; Vaginal delivery; Induced labor; There is no recommended mode of delivery

18. What is the recommended location of birth for a baby with gastroschisis? (select one)

Deliver at home; Delivery at a local health center; Delivery at any regional referral hospital; Delivery at a referral hospital with pediatric surgery services

19. What is the purpose of positioning a baby with gastroschisis on their right side a nasogastric tube in the first week for a baby with gastroschisis? (select all that apply)

To decrease the risk of aspiration and pneumonia; To facilitate gradual reduction of bowel into the abdomen; To provide hydration food; Protect bowel from twisting; Reduce water loss and dehydration; Reduce heat loss

20. What is the recommended positioning for a baby with gastroschisis? (select one)

Prone (laying on their belly); Left side down; Right side down; Supine (laying on their back)

21. What can happen if the intestines remain exposed without a covering? (select one)

Increased risk of ischemia and infection; Risk of traumatic perforation increases; The baby will not need surgery; Bowel will automatically reduce into the abdominal cavity

22. What long-term outcomes can occur in gastroschisis survivors? (select all that apply)

Heart defects; Bowel obstruction; Increased intelligence; Normal growth patterns

23. What should parents be educated about regarding gastroschisis? (select all that apply)

It is a curse; It is a manageable condition; Surgery is always required; Babies with this condition do not survive

24. What is the immediate role of fluid resuscitation in managing a baby with gastroschisis? (select one)

To promote bowel function; To prevent dehydration and electrolyte imbalance; To encourage feeding; To reduce hospital stay

25. Why should a baby with gastroschisis be kept immediately NPO? (select all that apply)
To prepare for surgery; To allow for bowel decompression; To promote breastfeeding; To reduce fluid intake; To reduce risk of perforation

26. What is a potential outcome for males who survive gastroschisis? (select one)
Infertility; Undescended testes; Increased cancer risk; Big testicle and scrotum; Big belly

27. What dietary changes can help reduce the risk of gastroschisis?

Increased fat intake; Higher intake of fruits and vegetables; Decreased protein intake; High sugar consumption

28. What is the goal of educating parents about gastroschisis? (select one)

To discourage hospital visits; To instill fear about the condition; To promote understanding and hope; To recommend home remedies

29. What is the typical birth weight concern for neonates with gastroschisis? (select one)
They are often of normal weight; They are usually low birth weight; They are often high birth weight; Weight is not a concern

30. Why is it important to keep the exposed bowel moist? (select one)

To facilitate faster growth; To prevent dehydration and damage; To enhance visibility for monitoring the bowel; To prevent bacterial growth

31. Which of the following are true? (select all that apply)

Pregnant mothers with a gastroschisis baby are at increased risk of preterm labor; Gastroschisis can run in a family (through genetic inheritance); Gastroschisis is caused by an intrauterine vascular injury the risk of which is increased by certain lifestyle factors in younger mothers and fathers; Gastroschisis is preventable

32. What is a common misconception about gastroschisis in many communities? (select all that apply)

It can be prevented; It is passed down in a family lineage; It is caused by environmental factors; It is a result of witchcraft.

33. What condition is commonly associated with omphalocele but not with gastroschisis? (select one)

Intestinal atresia; Cardiac abnormalities; Cryptorchidism (undescended testis)

34. What can be done to prevent bowel constriction in a baby with gastroschisis? (select one)

Avoid surgery; Enlarge the defect if necessary; Start oral feeding immediately; Use a tight bandage

35. What is the expected duration for recovery before a baby with gastroschisis can go home from the hospital? (select one)

1-2 days; 5-10 days; 3-4 months; 2-6 weeks; 5-7 months

36. What is the main role of antibiotics in managing gastroschisis? (select one)

To prevent sepsis and infection; To reduce inflammation; To promote feeding; To encourage bowel movement

37. What is a key determinant of improved outcomes/prognosis for babies with gastroschisis? (select one)

Birth weight over 5 kg; Receiving timely and appropriate medical care; Receiving spiritual cleansing; Being born at home

38. What is the recommended postnatal management for the umbilical cord stump? (select one)

Cut it short immediately; Cut it long for later use; Tie it tightly; Remove it as soon as possible

39. What maternal condition is NOT a risk factor for gastroschisis? (select one)

Maternal malnutrition during pregnancy; Nulliparity (never given birth to a child); Advanced maternal age; Caffeine intake during pregnancy

40. Which maternal behavior is linked to increased risk of gastroschisis? (select one)

Regular exercise; Tobacco smoking; Healthy eating; Attending prenatal/antenatal care

41. What is an important aspect of postnatal management (that is care after delivery) for the baby? (select one)

Encourage immediate oral feeding (breastfeeding); Maintain normal body temperature; Delay antibiotic administration; Don't touch the intestines.

42. What is the main factor contributing to the higher mortality rate of gastroschisis in low-income countries? (select one)

Genetic predisposition; Lack of timely medical care; Maternal age; Environmental toxins

43. What aspect of care is needed for reducing heat loss in gastroschisis? (select one)

Placing the baby in direct sunlight; Using a cold compress; Warming the intestines directly using candle or lamp; Covering the baby in clean dry clothes and covering the intestines with plastic wrap

44. What does a high level of maternal serum alpha-fetoprotein suggest? (select one)

Normal pregnancy; Potential abnormalities such as gastroschisis; Low birth weight; High fetal movement

45. What is the earliest time when gastroschisis can be diagnosed during pregnancy? (select one)

Between 8-10 weeks of pregnancy; After 12 weeks of pregnancy; After 25 weeks of pregnancy; After 17 weeks of pregnancy; At 6 weeks of pregnancy

46. What is the significance of the umbilical cord insertion point in diagnosing gastroschisis? (select one)

It is irrelevant to diagnosis; It helps differentiate between gastroschisis and omphalocele; It determines the type of delivery; It predicts the child's future health

47. Why do babies with gastroschisis require early medical/surgical intervention? (select all that apply)

They are always born at full term; They are more likely to be preterm and low birth weight; They do not need monitoring; They are less likely to survive without appropriate medical/surgical care

48. What impact does timely care have on outcomes for babies with gastroschisis? (select all that apply)

It is not significant; It reduces the chance of survival; It improves long-term quality of life; It increases the risk of complications; It improves the chance of survival

49. What should be communicated to parents about their child's condition? (select one)

There is no hope for recovery; The baby will require special schooling; Gastroschisis is survivable with appropriate care; They should not seek medical help

50. What is the mortality rate for babies with gastroschisis in high-income countries? (select one)

Less than 10%; Approximately 20%; About 50%; Over 90%

51. How comfortable are you in caring for babies with gastroschisis prior to transferring to a pediatric surgeon? (select one)

Extremely uncomfortable; Uncomfortable; Neutral; Comfortable; Extremely comfortable

Program Evaluation

1. Rate your satisfaction with the lectures. (select one)
   Extremely Unsatisfied; Unsatisfied; Neutral; Satisfied; Extremely Satisfied

2. How beneficial/helpful was this training course to you? (select one)
   Extremely unhelpful; Unhelpful; Neutral; Helpful; Extremely helpful

3. What language should be used to deliver this course in the future to a larger group?
   English; Luganda; Other (please specify)

4. How likely are you to teach other health workers all you have learned today? (select one)
   Extremely unlikely; Unlikely; Neutral; Likely; Extremely likely

5. If I were to teach other healthcare workers the principles in this course, I would feel comfortable to teach (select all that apply).
   Nurses; Midwives; Doctors (Health Officers, Residents, General Surgeons); Other (please specify)

6. Rate your satisfaction with the hands-on portion of the training. (select one)
   Extremely Unsatisfied; Unsatisfied; Neutral; Satisfied; Extremely Satisfied

7. What did you like about the training? Please explain....
8. What did you NOT like about the training? Please explain....
9. How can we make this training course better?
10. How confident are you in your ability to teach others ALL (lecture content and hands-on training) you have learned today? (select one)
    Extremely unconfident; Unconfident; Neutral; Confident; Extremely confident
